# HOXB7 promotes tumor progression via bFGF-induced activation of MAPK/ERK pathway and indicated poor prognosis in hepatocellular carcinoma

**DOI:** 10.18632/oncotarget.17004

**Published:** 2017-04-10

**Authors:** Wei-Min Wang, Yang Xu, Yao-Hui Wang, Hai-Xiang Sun, Yun-Fan Sun, Yi-Feng He, Qing-Feng Zhu, Bo Hu, Xin Zhang, Jing-Lin Xia, Shuang-Jian Qiu, Jian Zhou, Xin-Rong Yang, Jia Fan

**Affiliations:** ^1^ Department of Liver Surgery, Liver Cancer Institute, Zhongshan Hospital, Fudan University, Key Laboratory of Carcinogenesis and Cancer Invasion, Ministry of Education, Shanghai 200032, P. R. China; ^2^ Institute of Biomedical Sciences, Fudan University, Shanghai 200032, P. R. China; ^3^ Department of Interventional Radiology, Fudan University Shanghai Cancer Center, Department of Oncology, Shanghai Medical College, Fudan University, Shanghai 200032, P. R. China

**Keywords:** HOXB7, bFGF, hepatocellular carcinoma, prognosis, invasion and metastasis

## Abstract

The homeobox-containing gene HOXB7 plays an important role in the pathogenesis and progression of many cancers, yet its role in hepatocellular carcinoma (HCC) remains unclear. This study comprehensively analyzed the expression and clinical significance of HOXB7 in HCC and explored its potential mechanism in tumor progression. We found HOXB7 was highly expressed in HCC cell lines with highly metastatic potential and cancerous tissues from patients with tumor recurrence. The abilities of proliferation, migration, and invasion were notably decreased by depletion of HOXB7, and were enhanced by its enforced expression *in vitro*. HOXB7 expression was positively correlated with tumor progression and lung metastasis *in vivo*. The gene microarray data implied that HOXB7 affects biological functions of HCC cells through MAPK/ERK pathway activation. Further study confirmed that the effect of HOXB7 in activating MAPK/ERK pathway via induction of basic fibroblast growth factor (bFGF) secretion, and the inhibition of bFGF secretion could abolish MAPK/ERK pathway activation after ectopic expression of HOXB7. Chromatin immunoprecipitation experiments and luciferase reporter assays confirmed that HOXB7 promoted bFGF secretion via binding its promoter directly. Furthermore, the clinical significance of HOXB7 expression was confirmed using tissue microarrays containing 394 HCC tissue specimens. Patients with high HOXB7 expression showed shorter survival times and higher recurrence rates, and HOXB7 was an independent indicator for survival and recurrence. Overall, HOXB7 promotes HCC cell proliferation, migration, and invasion through the bFGF-induced MAPK/ERK pathway activation. It might be a novel prognostic factor in HCC and a promising therapeutic target for tumor metastasis and recurrence.

## INTRODUCTION

Hepatocellular carcinoma (HCC) is one of the most common malignancies worldwide [1]. Although long-term survival of HCC patients has been obtained in some clinical centers, the prognosis of HCC patients is still dismal due to the rapid progression of this disease [2]. The biologic and clinical behaviors of cancers are affected by multiple molecular pathways, which are mainly under the control of transcription factors [3]. Thorough exploration of how transcription factors affect cancer biology may promote understanding of the mechanisms of tumor development and progression and advance the discovery of novel therapeutic strategies [4].

HOX genes, a highly conserved subgroup of the homeobox superfamily, play crucial roles in development by regulating numerous processes including apoptosis, receptor signaling, differentiation, motility, and angiogenesis. Aberrations in HOX gene expression have been associated with abnormal development and malignancy [5–7]. As a member of HOX gene family, the roles of HOXB7 in tumorigenesis have been reported in several tumor types, including leukemias, ovarian carcinoma, breast cancer, gastric cancer and colorectal cancer [8–13]. Further studies indicated that HOXB7 induces epithelial mesenchymal transition (EMT), [9] transactivates pathways involving Ras/Rho, [9] PI3K/AKT, and MAPK [8]. These results suggest that HOXB7 plays an important role in tumor development and progression. Up to now, only one study revealed HOXB7 expression was significantly higher in HCC tissues, and it was an independent prognosis factor for OS [14], but the mechanism of HOXB7 involvement in HCC progress has not been elucidated.

Our preliminary data found that HOXB7 was highly expressed in HCC tissues compared with adjacent non-cancerous tissues based on transcript profiles, suggesting that HOXB7 might play a crucial role in HCC carcinogenesis and be worth further exploring. In this study, we comprehensively analyzed the expression and clinical significance of HOXB7 in HCC and explored its potential mechanism in tumor progression. We demonstrated that HOXB7 enhances proliferation, migration, and invasion of HCC cells through bFGF-induced MAPK/ERK pathway activation, and its expression was strongly associated with tumor recurrence and poor prognosis of HCC patients after surgery.

## RESULTS

### HOXB7 expression level is positive associated with the metastatic potential of HCC cell lines and tumor recurrence

**Figure 1:**
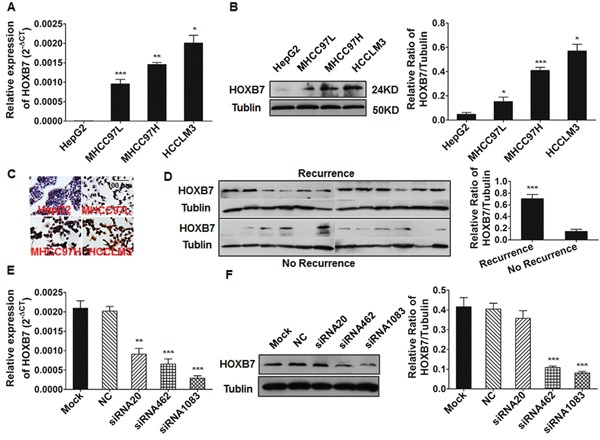
The expression of HOXB7 in HCC cell lines and tumor tissues **(A-C)** qRT-PCR, Western blot and immunocytochemical analysis of HOXB7 mRNA levels and protein expression. **(D)** Patients suffering HCC recurrence exhibited higher HOXB7 protein levels than those without recurrence. **(E, F)** HOXB7 mRNA and protein expression in HCCLM3 cells treated with siRNA for 48 and 72 hours was analyzed by qRT-PCR and Western blot respectively. *, *P*<0.05; **, *P*<0.01; ***, *P*<0.001.

HCC cells with higher metastatic potential expressed significantly higher levels of HOXB7 mRNA and protein than the less metastatic ones (Figure 1A, 1B). Immunocytochemical analysis showed that HOXB7 protein was expressed predominantly in the nuclei of high metastatic potential HCC cells (MHCC97H and HCCLM3 cells) (Figure 1C), and the staining intensity was consistent with qRT-PCR and western blot results. These results demonstrate that HOXB7 expression was positively correlated with the metastatic potential of HCC cell lines. Moreover, western-blot analysis in tumor tissues revealed that patients with tumor recurrence exhibited higher HOXB7 protein expression than those without recurrence (*P<0.001*, Figure 1D).

### HOXB7 promotes HCC cell proliferation, migration, and invasion *in vitro*

**Figure 2:**
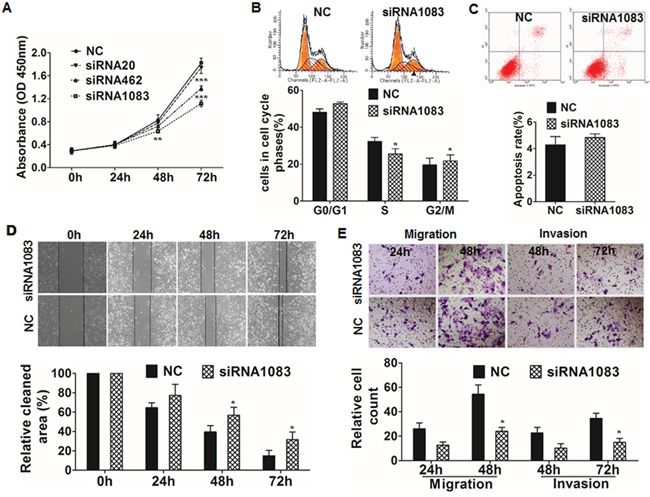
Functional analysis of HOXB7 by siRNA inhibition **(A)** CCK8 assay showed that cell proliferation was significantly suppressed after HOXB7 siRNA1083 transfection for 48 and 72 hours. **(B)** Cell cycle analysis showed that HOXB7 siRNA1083 induced S phase arrest and increased the proportion of cells in the G0/G1 phase. **(C)** Treatment with siRNA1083 had no significant effect on apoptosis in HCCLM3 cells. **(D)** Wound healing assays revealed a significant delay in the wound closure rate of HCCLM3 siRNA1083 cells compared with control cells at 48 and 72 hours after incubated with 25 μg/ml mitomycin C (Sigma, M4287-2MG) for 3h. **(E)** *In vitro* migration and invasion assays showed that the migration and invasion of the HOXB7 siRNA1083-treated group was significantly lower than that of the control group at 48 and 72 hours respectively. *, *P*<0.05; **, *P*<0.01; ***, *P*<0.001.

The expression of HOXB7 was measured by qRT-PCR and western blot in HCCLM3 cells after treatment with HOXB7 siRNA or in MHCC97L-HOXB7 pCDNA3 cells. HOXB7 expression was significantly inhibited after siRNA treatment (especially siRNA1083) and increased after ectopic introduction of HOXB7 (Figure 1E-1F, and Supplementary Figure 1A). The proliferation of HCCLM3 cells significantly decreased after treatment with HOXB7 siRNA, whereas proliferation of MHCC97L was significantly increased after HOXB7 up-regulation (Figure 2A and Supplementary Figure 1B). Cell cycle analysis showed that HOXB7 siRNA caused S phase arrest (25.48±2.91% *vs*. 32.29±2.18%, *P*<0.05) and accumulation of cells in G0/G1 phase (52.73±0.96% *vs*. 48.10±1.82%, *P*<0.05) compared with NC group (Figure 2B). Annexin V/PI staining showed that the apoptosis ratio was not significantly different between the HOXB7 siRNA and NC groups (*P*>0.05, Figure 2C). The wound-healing assay indicated that cell migration was significantly suppressed in HCCLM3 cells after siRNA treatment with 25 μg/ml mitomycin C (*P*<0.01, Figure 2D). Transwell system assays showed that down-regulation of HOXB7 significantly suppressed the migration and invasion abilities of HCCLM3 (*P*<0.05, Figure 2E). In contrast, the proliferation, migration, and invasion potentials of MHCC97L were significantly enhanced by HOXB7 overexpression (*P*<0.05, Supplementary Figure 1B, 1D and 1E). Cell cycle analysis showed that HOXB7 overexpression reduced the number of cells in S phase and induced G0/G1 phase arrest (*P*<0.05, Supplementary Figure 1C).

### HOXB7 promotes HCC progression and metastasis *in vivo*

**Figure 3:**
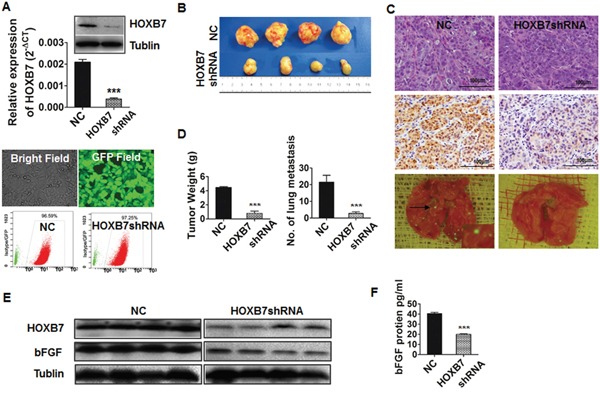
Down-regulation of HOXB7 suppress tumor growth and metastasis *in vivo* **(A)** HOXB7 expression in HCCLM3-pGCSIL-GFP and HCCLM3-pGCSIL-GFP-HOXB7 shRNA cells was measured by qRT-PCR, western blot analysis and immunofluorescence, flow cytometry assays. **(B)** Morphologic characteristics of tumors in the two groups. **(C)** Representative images of tumor specimens, HOXB7 expression by immunohistochemistry, and lung metastases by immunofluorescence in the two groups. **(D)** Tumor weight and lung metastatic lesions were significantly different between the two groups. **(E)** Decreased expression of HOXB7 and bFGF proteins in HOXB7 shRNA tumor tissues, and **(F)** lower serum bFGF level of mice in this group. ***, *P*<0.001.

HCC cell lines with stable down-regulation of HOXB7 expression (HCCLM3-pGCSIL-GFP-HOXB7 shRNA) were identified by qRT-PCR, immunoblotting, immunofluorescence and flow cytometry (Figure 3A). Comparison of the tumors induced in mice injected with cells expressing HOXB7 shRNA and control cells is shown in Figure 3B, and representative images of tumor, lung metastasis, and HOXB7 expression by immunohistochemistry are shown in Figure 3C. Tumor weights were significantly lower in the HOXB7 shRNA group than in the control group (0.775±0.330 g *vs*. 4.475±0.126 g, *P*<0.001, Figure 3D) and the formation of lung metastatic lesions was markedly decreased in the HOXB7 shRNA group (2.750±0.957 *vs*. 21.500±4.123, *P*<0.001, Figure 3D).

Conversely, tumor weights were significantly greater in the HOXB7 overexpression group (MHCC97L-HOXB7 pCDNA3) than in controls (2.083±0.581 g *vs*. 1.033±0.476 g, *P*<0.01, Supplementary Figure 2A, 2C) and the lung metastatic ratio was significantly increased (30% *vs*. 0%, *P*<0.01, Supplementary Figure 2B, 2C).

### Gene expression profile based on microarray analysis

**Figure 4:**
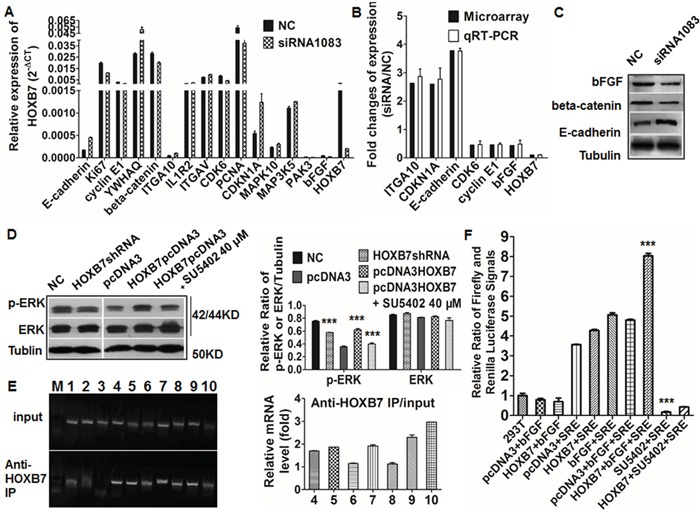
Validation gene changes after HOXB7 siRNA and its interaction with bFGF (**A** and **B**) Microarray experiments identified several genes that were differentially expressed between the siRNA group and the control group, and these findings were validated by qRT-PCR. **(C)** Expression of bFGF, beta-catenin, and E-cadherin in the siRNA and control group by western blot. **(D)** Expression of p-ERK was up-regulated in MHCC97L-HOXB7 pCDNA3 cells and down-regulated in HCCLM3-pGCSIL-GFP-HOXB7 shRNA cells, and SU5402 was down-regulated the expression of p-ERK in MHCC97L-HOXB7 pCDNA3 cells. **(E)** bFGF promoter-specific PCR primers could amplify this promoter region from DNA that was immunoprecipitated with the anti-HOXB7 antibody but not with the nonimmune IgG. **(F)** HOXB7 and bFGF significantly increased SRE luciferase reporter activity, while SU5402 could restrain it. (***, *P*<0.001).

Microarray analysis showed that 291 genes were differentially expressed between HOXB7 siRNA-treated HCCLM3 cells and the NC-treated group. Of these, 130 genes were up-regulated (ratio >1.5), and 161 genes were down-regulated (ratio <0.67). Many of these genes, including those encoding bFGF, MAPK10, MAP3K5, IL1R2, E-cadherin, Ki67, Cyclin E1, beta-catenin, 14-3-3 protein T-cell (YWHAQ), integrin alpha-V (ITGAV), integrin alpha-10 (ITGA10), CDK6, PCNA, CDKN1A, and PAK3, are related to tumor metastasis and regulation of cell migration (Figure 4A) [15]. Using the Kyoto Encyclopedia of Genes and Genomes, BioCarta, and Gene Map annotator and Pathway Profiler databases, the significant signaling pathways were categorized into groups that included MAPK signaling, Wnt signaling, p53 signaling, focal adhesion, and cell cycle (Supplementary Table 1). These pathways have previously been associated with carcinogenesis and metastasis [15]. Supplementary Table 1 lists the 10 pathways that are most likely to be involved in these processes. Our microarray results showed significant changes in the expression of a large number of genes involved in the MAPK pathway, such as *bFGF*, *MAPK10*, *MAP3K5*, *IL1R2*, *DUSP4*, *CDC42*, *MAP3K7IP2*, *TGFB1*, *FGF13*, *FOS*, *RASA1*, and *PRKCB1*.

Differential expression of the following genes was validated by qRT-PCR: E-cadherin, YWHAQ, ITGAV, ITGA10, CDKN1A, IL1R2, MAPK10, MAP3K5, and PAK3 (up-regulated), and bFGF, Ki67, Cyclin E1, beta-catenin, CDK6, and PCNA (down-regulated). The qRT-PCR results corresponded well with the microarray data (Figure 4B). Expression of bFGF, beta-catenin, and E-cadherin was also validated by western blotting (Figure 4C).

### HOXB7 enhances proliferation, migration, and invasion of HCC cells through activation of the MAPK/ERK signaling pathway via induction of bFGF secretion

**Figure 5:**
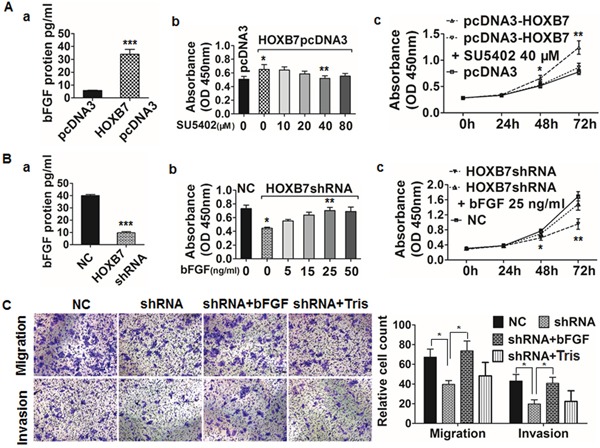
HOXB7 enhances HCC cell proliferation, migration, and invasion via induction of bFGF secretion **(A)** The level of bFGF secreted by MHCC97L-HOXB7 pCDNA3 cells was higher than that in the control group. The proliferation of HOXB7 pCDNA3 cells significantly increased than the control group at 48 hours. SU5402 inhibited the proliferation especially at 40 μM. The cell proliferation was significantly inhibited using 40 μM SU5402 for 48 hours and 72 hours in HOXB7 pCDNA3 cells. **(B)** The decreased bFGF level was detected in supernatant of HCCLM3-pGCSIL-GFP-HOXB7 shRNA cells, the proliferation of HOXB7 shRNA cells decreased significantly than the control at 48 hours and bFGF increased the proliferation especially at 25 ng/ml, the cell proliferation ability was significantly enhanced after adding 25 ng/ml bFGF to HOXB7 shRNA cells for 48 and 72 hours. **(C)** *In vitro* migration and invasion assays showed a significant decrease in the HCCLM3 HOXB7 shRNA group compared with the control group at 48 and 72 hours, respectively, and was partially reversed by adding 25 ng/ml bFGF to the upper chamber, not by 5mM Tris (bFGF supplementation). (*, *P*<0.05; **, *P*<0.01; ***, *P*<0.001).

As the most down-regulated molecule involved in MAPK pathway (the top one changed pathways significantly after HOXB7 siRNA treatment, Supplementary Table 1), the effect of bFGF on HCC and its relationship with HOXB7 was further explored. We found that bFGF expression was consistent with the HOXB7 expression level in HCC cell lines (Figure 4A-4C). As a secreted protein, bFGF level in cell supernatant was elevated significantly after ectopic expression of HOXB7 in MHCC97L cells (*P*<0.001, Figure 5Aa) and dramatically suppressed in HOXB7 shRNA-treated HCCLM3 cells (*P*<0.001, Figure 5Ba).

*In vivo*, tumor tissues of MHCC97L-HOXB7 pCDNA3 mice showed higher expression levels of bFGF protein than the control group (Supplementary Figure 2D). Likewise, bFGF levels in the serum of MHCC97L-HOXB7 pCDNA3 mice were higher than in the control group by ELISA (*P*<0.001, Supplementary Figure 2E). The same trend was observed in the HCCLM3 HOXB7 shRNA group and the control by western blot and ELISA analyses (*P*<0.001, Figure 3E, 3F). Meanwhile, we also found that human tumor tissues with high HOXB7 expression showed higher bFGF expression (Supplementary Figure 3B, 3C). A scatter plot of HOXB7 and bFGF expression in 50 HCC cancer tissues revealed a significant positive correlation (r^2^=0.4416, P<0.001, Supplementary Figure 3D).

We further investigated the role of bFGF in tumor cell proliferation, migration, and invasion. The proliferation ability of MHCC97L-HOXB7 pcDNA3 cells was significantly decreased after treatment with 40μM SU5402 for 48 and 72 hours, (Figure 5Ab, 5Ac). Addition of 25 ng/ml bFGF to the supernatant of HCCLM3 HOXB7 shRNA cells for 48 and 72 hours significantly increased cell proliferation (Figure 5Bb, 5Bc). To analyze the role of bFGF in invasion, HCCLM3 HOXB7 shRNA transfectants were incubated with 25 ng/ml bFGF in the upper chamber of a transwell plate for 72 hours. Metastasis assays were performed as for the invasion assays except without Matrigel. The presence of bFGF significantly increased the numbers of HCCLM3 HOXB7 shRNA cells exhibiting 48-hour migration and 72-hour invasion (*P*<0.05, Figure 5C).

As the top one significantly changed pathways after HOXB7 siRNA treatment, MAPK pathway might be an important one in the regulation of tumor progression by HOXB7. We further investigated the downstream genes in the MAPK pathway and found that levels of p-ERK, a member of the MAPK family, were increased in MHCC97L-HOXB7 pCDNA3 and reduced in HCCLM3 HOXB7 shRNA compared with respective controls. It implied that expression of p-ERK is positively correlated to the HOXB7 level. Addition of 40 μM SU5402 to the MHCC97L-HOXB7 pCDNA3 culture medium significantly decreased the expression of p-ERK expression but had no effect on expression of ERK (Figure 4D). Thus, we speculated that HOXB7 affects biological functions of HCC cells through the bFGF-induced MAPK/ERK pathway activation.

### HOXB7 activates MAPK/ERK signaling pathway via direct target bFGF in HCC cells

To further confirm the transcriptional regulation of the bFGF gene by HOXB7, we performed CHIP and luciferase reporter assays in HCCLM3 cells. We found that bFGF promoter-specific PCR primers amplified this promoter region from DNA that was immunoprecipitated with the anti-HOXB7 antibody but not with the nonimmune IgG (Figure 4E). These findings demonstrated that the HOXB7 protein could target and interacts with the promoter regions of bFGF in HCCLM3 cells, and then induced bFGF secretion through physically binding bFGF promoter.

We found that bFGF could increase the MAPK/ERK transcription factor (SRE) luciferase reporter activity, and this effect was enhanced or inversed when HOXB7 pcDNA3 or SU5402 was respectively added. bFGF and HOXB7pcDNA3 cotransfection could obviously increase the promoter luciferase reporters activity of SRE, while SU5402 with HOXB7pcDNA3 could restrain SRE (P<0.001, Figure 4F).

Luciferase reporter activity assays showed that HOXB7 overexpression and bFGF significantly increased MAPK/ERK reporter activity and the effect was blocked by treatment with the SU5402. These results suggested that bFGF can activate the MAPK/ERK promoter, and then induced the activation of MAPK pathway.

### High HOXB7 expression predicts poor prognosis in HCC patients

HOXB7 protein was predominantly detected in the nuclei of tumor cells, although cytoplasmic staining was also observed. The expression of HOXB7 in cancer tissues was classified as high (score ≥6) in 181 cases (45.9%) and low (score 0-5) in 213 cases (54.1%), whereas there were almost no cases of high HOXB7 expression in adjacent non-cancerous tissues (data not shown). Representative images and statistical data are shown in Supplementary Figure 3A and Table 1. High HOXB7 expression was significantly correlated with several clinicopathologic parameters, including multiple tumors (*P*=0.007), vascular invasion (*P*=0.010), satellite lesion (*P*=0.003), and Barcelona Clinic Liver Cancer (BCLC) Stage B+C (*P*=0.003) (Table 1).

**Table 1:** Correlation between the factors and clinicopathologic characteristics in 394 patients

Clinical and pathological indexes		HOXB7		
High	Low	*P*
Age (years)	>50	86	108	
	≤50	95	105	0.528
Sex	Male	163	181	
	Female	18	32	0.131
AFP (ng/ml)	>20	120	133	
	≤20	61	80	0.426
HBsAg	Positive	153	170	
	Negative	28	43	0.225
HCV	Positive	6	3	
	Negative	175	210	0.312^Δ^
GGT (U/l)	>54	113	118	
	≤54	68	95	0.158
ALT (U/l)	>75	18	19	
	≤75	163	194	0.728
Liver cirrhosis	Yes	153	167	
	No	28	46	0.121
Tumor size	>5	82	84	
	≤5	99	129	0.240
Tumor number	Multiple	47	32	
	Single	134	181	**0.007**
Vascular invasion	Yes	56	42	
	No	125	171	**0.010**
Tumor encapsulation	None	78	85	
	Complete	103	128	0.522
Satellite lesion	Yes	25	11	
	No	202	156	**0.003**
Edmondson Stage	III-IV	73	62	
	I-II	119	140	0.997
Child-Pugh Stage	B	6	13	
	A	175	200	0.198
BCLC Stage	B+C	52	35	
	0+A	129	178	**0.003**

Abbreviations: AFP, alpha-fetoprotein; GGT, gamma glutamyl transferase; BCLC, Barcelona Clinic Liver Cancer; HBsAg, hepatitis B surface antigen; HCV, hepatitis C virus;

^Δ^ Fisher's exact tests; Chi-square tests for all the other analyses.

**Figure 6:**
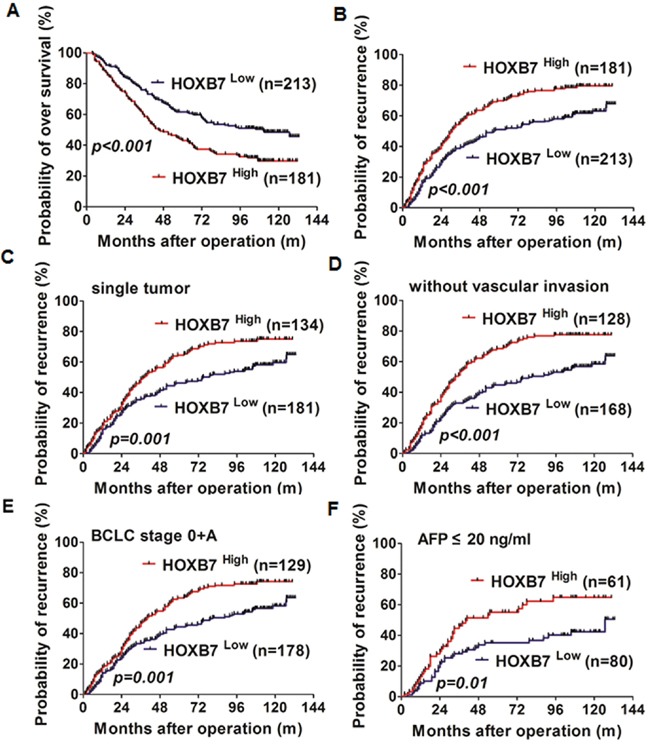
HOXB7 and prognosis of HCC **(A, B)** Kaplan-Meier analysis of OS and cumulative recurrence rates in 394 cases based on HOXB7 expression. Prognostic role of HOXB7 in patients with single tumor **(C)**, without vascular invasion **(D)**, BCLC stage 0+A **(E)**, and AFP ≤ 20 ng/ml **(F)**.

At the last follow-up, 49.5% (195/394) of the patients had suffered a recurrence and 41.6% (164/394) had died. The 1-, 3-, 5- and 8-year OS rates were 88.6%, 66.5%, 51.5%, and 42.9%, respectively, and corresponding cumulative recurrence rates were 30.0%, 54.1%, 59.4%, and 72.1%. In univariate analysis, alpha-fetoprotein (AFP) level, gamma-glutamyl transpeptidase (GGT) level, tumor size, tumor number, vascular invasion, tumor encapsulation, satellite lesion, BCLC stage, and HOXB7 expression were unfavorable predictors for OS and/or TTR (except tumor size) (Table 2). HOXB7 was found be prognostic for OS (HR=1.719, 95% confidence interval (CI) 1.325-2.229, *P*<0.001) and TTR (HR=1.645, 95%CI 1.294-2.092, *P*<0.001) (Figure 6A, 6B). Further multivariate analysis indicated that HOXB7 was an independent prognostic factor for OS (HR=1.188, 95%CI 1.039-1.359, *P*=0.012) and TTR (HR=1.413, 95%CI 1.102-1.813, *P*=0.006) (Table 2). We further investigated the predictive value of HOXB7 within clinical subgroups of early-stage, well-differentiated, and normal AFP (≤20 ng/ml). The prognostic significance of HOXB7 persisted in HCC patients with a single tumor (*P*=0.001), without vascular invasion (*P*<0.001), BCLC stage 0+A (*P*=0.001), with normal AFP levels (*P*=0.01), small tumor size (diameter ≤5 cm) (*P*=0.01), without satellite lesion (*P*<0.001) and with well-differentiated tumor (Edmonson stage I-II) (*P*<0.01) (Figure 6C-6F and Supplementary Figure 4).

**Table 2:** Univariate and multivariate analyses of prognostic factors in 394 HCC patients

Variables	OS		TTR	
	HR (95% CI)	*P*	HR (95% CI)	*P*
**Univariate analysis**				
Age (years)( >50 vs. ≤50)	0.584(0.718-1.205)	0.584	0.927(0.730-1.177)	0.927
Sex (Male vs. Female)	1.206(0.796-1.827)	0.377	1.147(0.789-1.667)	0.474
AFP (ng/ml) (>20 vs. ≤20)	1.610(1.214-2.135)	**0.001**	1.367(1.061-1.760)	**0.016**
HBsAg (Positive vs. Negative)	1.417(0.989-2.031)	0.058	1.313(0.948-1.820)	0.102
HCV (Positive vs. Negative)	1.094(0.486-2.462)	0.828^Δ^	0.899(0.400-2.019)	0.796^Δ^
GGT (U/l) (>54 vs. ≤54)	1.644(1.252-2.159)	**<0.001**	1.538(1.200-1.972)	**0.001**
ALT (U/l)( >75 vs. ≤75)	0.919(0.581-1.454)	0.718	0.809(0.528-1.241)	0.333
Tumor size (cm) ( >5 vs. ≤5)	1.461(1.126-1.894)	**0.004**	1.224(0.961-1.558)	0.101
Tumor number(Multiple vs. Single)	2.082(1.550-2.796)	**<0.001**	2.210(1.671-2.923)	**<0.001**
Vascular invasion (Yes vs. No)	1.793(1.351-2.379)	**<0.001**	1.761(1.350-2.296)	**<0.001**
Tumor encapsulation(None vs. Complete)	1.427(1.100-1.850)	**0.007**	1.331(1.046-1.693)	**0.020**
Satellite lesion (Yes vs. No)	1.651(1.089-2.502)	**0.018**	1.639(1.101-2.441)	**0.015**
Edmondson Stage (III-IV vs. I-II)	1.068(0.814-1.401)	0.636	1.057(0.823-1.358)	0.662
Child-Pugh (B vs. A)	1.339(0.765-2.345)	0.307	1.434(0.865-2.379)	0.162
BCLC Stage (C+D vs. A+B)	2.601(1.960-3.451)	**<0.001**	2.663(2.034-3.486)	**<0.001**
HOXB7 (High vs. Low)	1.719(1.325-2.229)	**<0.001**	1.645(1.294-2.092)	**<0.001**
**Multivariate analysis**				
Tumor size (cm) ( >5 vs. ≤5)	1.352(1.023-1.787)	**0.034**	n.a.	
Tumor number(Multiple vs. Single)	1.660(1.191-2.314)	**0.003**	1.882(1.375-2.575)	**<0.001**
Vascular invasion(Yes vs. No)	1.428(1.056-1.931)	**0.021**	1.384(1.046-1.832)	**0.023**
Tumor encapsulation(None vs. Complete)	1.130(0.853-1.498)	0.393	1.046(0.808-1.353)	0.735
Satellite lesion (Yes vs. No)	0.898(0.566-1.425)	0.649	0.875(0.561-1.365)	0.557
HOXB7(High vs. Low)	1.188(1.039-1.359)	**0.012**	1.413(1.102-1.813)	**0.006**

NOTE: ^Δ^Fisher's exact tests; Chi-square tests for all the other analyses.

Abbreviations: OS, overall survival; TTR, time to recurrence; HR, hazard ratio; 95% CI, 95% confidence interval; AFP, alpha-fetoprotein; HBsAg, hepatitis B surface antigen; HCV, hepatitis C virus; GGT, gamma glutamyl transferase; ALT, alanine aminotransferase; BCLC, Barcelona Clinic Liver Cancer; TNM, tumor-node-metastasis; n.a., not adopted.

## DISCUSSION

It is very important to identify the factors associated with tumor recurrence and metastasis because these are the most common causes of mortality after surgical treatment of HCC [16]. In this study, we showed that the homeobox protein HOXB7 was overexpressed in HCC tissues compared with adjacent non-cancerous tissues, in highly metastatic HCC cell lines, and in patients with tumor recurrence, but was barely detected in low-metastatic HCC cells or HCC tissues of patients without recurrence. A positive correlation between HOXB7 expression and multiple tumors, satellite lesions, vascular invasion, and more advanced tumor stage indicated that HOXB7-positive tumor cells have a more aggressive phenotype. *In vitro* depletion or overexpression experiments showed that HOXB7 promotes tumor cell proliferation, migration, and invasion in HCC. Further investigation indicated that HOXB7 is a potent inducer of bFGF secretion and activates the MAPK/ERK signaling pathway, which had previously been advocated as an important mechanism during HCC invasion transformation and metastasis [17]. The roles of HOXB7 in enhancing the proliferation of tumor cells, as well as promoting migration and invasion functions of cancer cells during hematogenous dissemination, are presumably responsible for the high recurrence rate and poor prognosis observed in HOXB7-positive HCC patients. Furthermore, from a therapeutic viewpoint our data indicate that molecular therapies targeting HOXB7 in HCC might be a promising approach to blocking tumor progression.

Our results confirmed HOXB7 as an independent significant risk factor for tumor recurrence and survival after curative resection, and it was in accordance with one recently study [14]. In clinical practice it is challenging to predict tumor relapse in HCC subgroups with a low risk of recurrence, such as single tumor, small tumor, without vascular invasion, absence of satellite lesion, BCLC stage 0+A, and well-differentiated tumor [18]. We found that HOXB7 retained prognostic value in these subpopulations. The predictive significance of HOXB7 in these subgroups would help clinicians identify patients at high risk of recurrence and enable them to administer rational adjuvant therapy after surgery. Currently, AFP is widely used to monitor recurrence and metastasis in AFP-positive HCC patients after surgery [19]. However, 40% to 60% of HCC patients exhibit normal AFP levels, and it is difficult to surveillance the metastasis and recurrence in those patients after resection [18, 20]. In this study, we found that 61 patients in the AFP-normal group (43.3%) expressed high levels of HOXB7, and the prognosis of these patients was dismal. The median TTR in HOXB7-high patients was 24 months, compared with 101.8 months in the HOXB7-low group, and most of the HOXB7-high patients (65.6%) died from HCC recurrence within 5 years. Thus, HOXB7 might be a useful predictor for HCC patients in subgroups for which prognosis is very difficult to predict using conventional clinical indexes.

Until now, the function of HOXB7 in HCC and the underlying mechanisms were not clear. Using a human whole genome oligomicroarray, we explored the potential molecular mechanism of HOXB7 by identifying genes that are differentially expressed between HCC cells treated with HOXB7 siRNA and those treated with scrambled siRNA. Our data indicated that HOXB7 participates in several signaling pathways involved in tumor development and progression, such as the MAPK pathway, Wnt signaling pathway, p53 signaling pathway, focal adhesion, and cell cycle. Among the 161 down-regulated genes, only bFGF has previously been documented to be involved in HOXB7 regulation [9]. Other candidate genes that appear to directly or indirectly regulate by HOXB7, such as those encoding Ki67, cyclin E1, beta-catenin, CDK6, and PCNA, have not been reported previously. Among the 130 up-regulated genes, those encoding E-cadherin, MAPK10, MAP3K5, and PAK3 have been confirmed to inhibit tumor proliferation or invasion [21–24]. Further bioinformatics analysis showed that a large number of genes involved in the MAPK pathway were differentially regulated, suggesting that the MAPK pathway might play an important role in the mechanism by which HOXB7 participates in HCC progression. Gene microarray data and qRT-PCR analysis confirmed that bFGF expression dramatically decreased after siRNA treatment of HCCLM3 cells (>2.0 fold) (Figure 4A-4C). The high expression of bFGF was observed in both MHCC97L-HOXB7 pCDNA3 cells and the corresponding xenograft tumors, while it was low in HCCLM3-pGCSIL-GFP-HOXB7 shRNA cells and tumors (Figure 3E-3F, 5A-5B and Supplementary Figure 2D-2E). A significant positive correlation between bFGF and HOXB7 expression was found in 50 HCC cancerous tissues (Supplementary Figure 3B-3D). Moreover, inhibition of the bFGF autocrine signaling cascade using the FGF receptor inhibitor SU5402 suppressed the proliferation of MHCC97L-HOXB7 pCDNA3 cells (Figure 5Ab), while recombinant human FGF-basic (bFGF) increased the proliferation, migration, and invasion of HCCLM3-pGCSIL-GFP-HOXB7 shRNA cells (Figure 5Bb c and 5C). Using CHIP and luciferase reporter genes assays, we found that HOXB7 can stimulate bFGF secretion through binding the bFGF promoter directly, then activate MAPK/ERK signaling, and this is the first demonstration for the interaction of HOXB7 and bFGF in HCC (Figure 4E, 4F). Further study showed that obstructing FGF signaling by SU5402 could attenuate activation of the ERK pathway in MHCC97L-HOXB7 pCDNA3, suggesting that the biological functions of bFGF in promoting tumor proliferation and invasion were through activating MAPK/ERK signaling (Figure 4D). Taking together, these data indicate that HOXB7 promotes HCC progression and metastasis through activating the secretion of bFGF and subsequently triggering MAPK/ERK pathways and ERK phosphorylation to regulate HCC proliferation, migration, and invasion. Further studies on HOXB7 target genes and their regulation will provide new insights into the underlying mechanisms.

Limitations of our study include its retrospective nature and the fact that most of the patients had a background of hepatitis B infection. Thus, the prognostic significance of HOXB7 needs further validation using larger and more diverse patient cohorts. Meanwhile, more intensive research into the molecular mechanism of HOXB7 functions in HCC need to be further undertaken.

In conclusion, this study demonstrates that HOXB7 promotes HCC cell proliferation, invasion, and metastasis through activation of the MAPK/ERK pathway by stimulating bFGF secretion. HOXB7 expression serves as a novel prognostic indicator for HCC patients undergoing curative resection. Cancer therapy targeted against HOXB7 might be a promising approach for the treatment of HCC metastasis and recurrence.

## MATERIALS AND METHODS

### Cells and transfection, expression, and functional assays

HepG2 and 293T cells used in Luciferase Reporter Assay were obtained from the Shanghai Institute for Biological Science (Shanghai, China). MHCC97L, MHCC97H, and HCCLM3 with stepwise metastatic potential were established at our institute [25]. They were routinely maintained in high-glucose DMEM with 10% FBS (Gibco). Three pairs of HOXB7 small interfering RNA (siRNA) oligos (siRNA20, sense GGACUCUAAUUCUGUAAUATT and antisense UAUUACAGAAUUAGAGUCCTT; siRNA462, sense GAGAGUAACUUCCGGAUCUTT and antisense AGAUCCGGAAGUUACUCUCTT; siRNA1083, sense GCUAUUGUAAGGUCUUUGUTT and antisense ACAAAGACCUUACAAUAGCTT) and a negative control (NC) (sense UUCUCCGAACGUGUCACGUTT and antisense ACGUGACACGUUCGGAGAATT) were synthesized by Genepharma and transfected into HCCLM3 cells using Lipofectamine 2000 (Invitrogen). HOXB7-pCDNA3 plasmid was kindly provided by Dr. Judith Gasson (University of California at Los Angeles, CA) [26] and pcDNA3 vector was purchased from Invitrogen. pGCSIL-HOXB7, a HOXB7-RNA interference (RNAi) lentiviral vector, was constructed by Shanghai GeneChem Co. Double-stranded oligonucleotides encoding human HOXB7-vshRNA (reverse primer: 5’-CCTCACGGAAAGACAGATCAATCTCTTGAATTGATCTGTCTTTCCGTGAGG-3’; forward primer: 5’-CCTCACGGAAAGACAGATCAATTCAAGAGATTGATCTGTCTTTCCGTGAGG-3’; target sequence: 5’-CCTCACGGAAAGACAGATCAA-3’) were annealed and inserted into the shRNA lentiviral expression vector pGCSIL-green fluorescent protein (GFP). pGCSIL-GFP alone was used as a NC. HOXB7-pCDNA3 and pGCSIL-GFP-HOXB7 shRNA were transfected into MHCC97L (to give MHCC97L-HOXB7 pCDNA3) and HCCLM3 (to give HCCLM3-pGCSIL-GFP-HOXB7 shRNA) using Lipofectamine 2000 and Polybrene (Sigma-Aldrich) respectively. pcDNA3 and pGCSIL-GFP vectors were used as controls (MHCC97L-pcDNA3 and HCCLM3-pGCSIL-GFP). MHCC97L-HOXB7 pCDNA3 and MHCC97L-pcDNA3 cells were selected in medium containing 0.7 mg/mL G418 (Sigma-Aldrich) for 5 weeks to establish stable neomycin-resistant clones. HCCLM3-pGCSIL-GFP-NC and HCCLM3-pGCSIL-GFP-HOXB7 shRNA cells were selected and confirmed by flow cytometry (BD Biosciences, CA, USA). All procedures were performed according to the manufacturers’ instructions. Expression levels of HOXB7 in transfected cells were confirmed by qRT-PCR, immunoblotting, immunofluorescence, or flow cytometric analysis. The methods were performed according to the manufacturer's recommendations and previously described [27, 28]. The details of primers used are given in Supplementary Table 2.

Cell proliferation, cell cycle distribution and cell apoptosis were detected using the Cell Counting Kit-8 (CCK-8) (Beyotime, China), Cell Cycle Analysis Kit (Beyotime) and Annexin V-FITC/PI Apoptosis Detection kit (BD Biosciences, CA, USA) respectively according to the manufacturer's protocol and previously described [29, 30]. Cell Migration, and Matrigel Invasion Assays were performed as previously described [9, 31–33].

### Animals and *in vivo* assays for tumor growth and distant metastasis

The procedures were performed as previous reports [29, 31, 32]. Briefly, Male athymic BALB/c nude mice (4-6 weeks old; Shanghai Institute of Material Medicine) were raised. 5×10^6^ cells were subcutaneously injected into the flanks of mice. After 5 weeks, mice were sacrificed, the tumors were harvested, serum and protein were extracted, and the weight of tumors and the metastatic lesions of lung were calculated as previously described [34]. Tumor tissue sections were prepared and HOXB7 immunoreactivity (Abnova, Taipei, China) was analyzed. All procedures were approved by The Animal Care and Use Committee of Fudan University, Shanghai, China.

### Microarray-based gene expression profile

A microarray assay was performed to compare the gene expression profiles between HOXB7 siRNA1083-treated and NC-treated HCCLM3 cells. The 22-K oligonucleotide microarrays were constructed by Capital Bio Corp (Beijing, China). Differences in gene expression profiles between the two groups were analyzed as previously reported [34]. To confirm the microarray results, several differentially expressed genes were further analyzed by qRT-PCR or western blotting.

### Enzyme-linked immunosorbent assay (ELISA)

bFGF levels in cell supernatants, mouse serum, and 50 cancerous tissues were determined using ELISA according to the manufacturer's protocol (R&D Systems) and previous studies [30].

### The effect of FGF receptor 1 (FGFR1) signaling pathway inhibitor and recombinant human bFGF in HCC cell lines

Cells were cultured in 96-well plates (3,000 cells/100 μl/well) for 12 hours and serum starved for 24 hours. Cells were treated with SU5402 (0, 10, 20, 40, and 80 μM) (Calbiochem, San Diego, CA), an inhibitor of FGFR1 signaling pathway, [35–37] or recombinant human bFGF (0, 5, 15, 25, and 50 ng/ml) (PeproTech, Rocky Hill, NJ) [12, 37] for 48 hours in DMEM containing 3% FBS. Concentrations of 40 μM SU5402 and 25 ng/ml bFGF were then selected for subsequent treatment of cells for 24, 48, and 72 hours. Cell proliferation was determined by CCK8 analysis.

### Chromatin immunoprecipitation (CHIP) and luciferase reporter assays

To elucidate the mechanism of HOXB7 regulates, CHIP and luciferase reporter assays were then performed as described previously [38]. HCCLM3 were fixed in 1% (vol/vol) formaldehyde for 10 minutes. Then fixation was quenched in 125 mmol/L glycine for 5 minutes. Nuclei were disrupted with 1% SDS lysis buffer for 15 minutes. Samples then were sonicated to shear DNA to 200~1000 bp. Supernatants obtained after centrifugation at 13,000×g for 10 min were used for immunoprecipitations using an anti-HOXB7 antibody (SC-133670 and SC-81292, Santacruz) or control IgG overnight at 4°C with rocking. The protein-DNA complex was collected with IgG beads (Roche). Crosslinking was reversed by incubation at 65°C for 1.5 hours and digested with Proteinase K (Sigma-Aldrich) at 45°C for 2 hours. DNA was purified using a Qiaquick PCR purification kit (Qiagen). The recovered DNA was resuspended in Tris-HCl EDTA buffer and used for the PCR amplification. Total cellular DNA was used as input control. The PCR primers for the target promoters (bFGF) are listed in Supplementary Table 3.

293T cells cultured in 96-well plates (40,000 cells/100 μl Opti-MEM/well) were cotransfected with 200 ng of HOXB7-pCDNA3 plasmid and SRE Reporter which represent MAPK/ERK Pathway (Cignal SRE Reporter (luc) Kit, SABiosciences) according to Cignal Reporter Assay Handbook. After 36 hours, to determine the effect of bFGF on these promoter activities, we treated the cells with 40 μM SU5402 for 4 hours or recombinant human bFGF (25 ng/ml) for half one hour. Then the ratios of Firefly to Renilla Luciferase activities were measured in cell lysates with the Dual Luciferase Reporter Assay System (Promega).

### Patients and follow-up

Fifty fresh HCC tissues were collected from HCC patients undergoing curative resection in the Liver Cancer Institute, Zhong Shan Hospital, Fudan University from 2010 to 2011, and were snap-frozen for ELISA analysis, and the corresponding paraffin-embedded tissues were used for immunohistochemistry staining. 12 frozen tissues were randomly chosen for western blot analysis from these cases.

Tumor specimens used in tissue microarrays (TMAs) analysis were consecutively chosen from 394 HCC patients who underwent liver resection in our institute between 2000 and 2002. All specimens were collected as described previously [27, 39]. The clinicopathologic characteristics are summarized in Supplementary Table 4. Survival data, including overall survival (OS) and time to recurrence (TTR), were collected until March 15, 2013. The median follow-up was 64 months (range, 3.5-133 months). Postoperative treatment modalities and surveillance followed uniform guidelines [39]. OS and TTR were defined as the interval between the date of surgery and death (or the last observation point taken), or any diagnosed relapse (intrahepatic recurrence and extrahepatic metastasis), respectively [31, 34, 40]. Approval for use of human subjects was obtained from the research ethics committee of Zhong Shan Hospital.

### Immunohistochemistry

TMAs were constructed as previously reported [28, 39]. Mouse monoclonal anti-human HOXB7 (1:1,000, Abnova) was used for immunohistochemistry with a two-step protocol [31, 39]. Scores reflected the intensity and percentage of positive staining tumor cell nuclei in the whole tissue cylinder as in our pervious study [34]. Overall scores <6 and ≥6 were defined as low and high expression respectively [34]. In studies investigating the relationship between bFGF and HOXB7, three representative fields were captured under high-power magnification (200×) for 50 paraffin-embedded tissues and HOXB7 density was determined using Image-Pro Plus v6.2 software as previously [31].

### Statistical analysis

Statistical analyses were performed with SPSS 19.0 for Windows (IBM) and GraphPad Prism 5 software as previously described [32]. The chi-square test, Fisher's exact test, and Student's t-test were used for comparison between groups as appropriate. OS and cumulative recurrence rates were calculated by the Kaplan-Meier method and analyzed by the log-rank test. Univariate and multivariate analyses were based on the Cox proportional hazards regression model. A *P*-value <0.05 was considered statistically significant.

## SUPPLEMENTARY MATERIALS FIGURES AND TABLES


