# Supplementary material for: HOXB7 promotes tumor progression via bFGF-induced activation of MAPK/ERK pathway and indicated poor prognosis in hepatocellular carcinoma
Source: Oncotarget. 2017 Apr 10;8(29):47121–35. doi: 10.18632/oncotarget.17004 (PMC5564549; doi:10.18632/oncotarget.17004)
Supplement: Supplementary file 1 [file oncotarget-08-47121-s001.pdf]

HOXB7 promotes tumor progression via bFGF-induced activation of MAPK/ERK pathway and indicated poor prognosis in hepatocellular carcinoma

SUPPLEMENTARY FIGURES AND TABLES

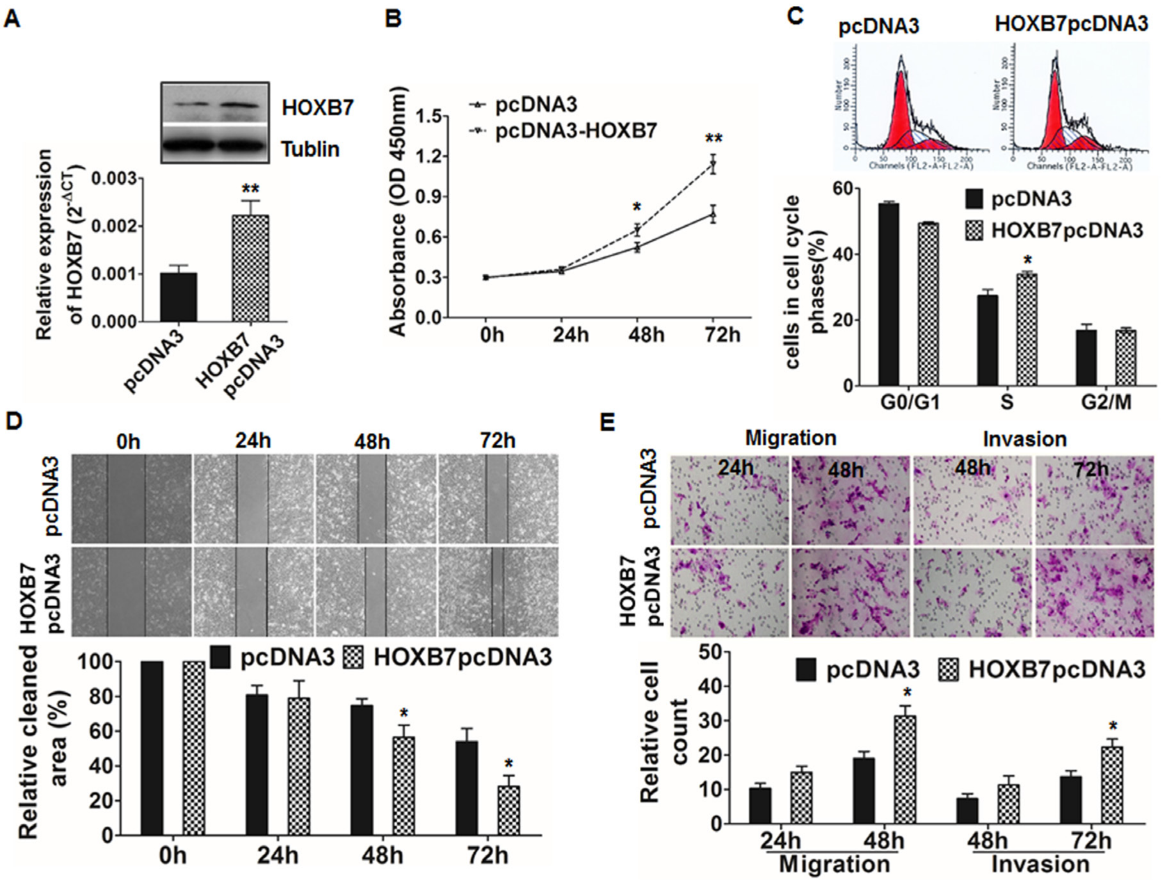

Supplementary Figure 1: HOXB7 expression in MHCC97L-HOXB7pCDNA3 and MHCC97L-pcDNA3 cells detected by qRT-PCR and western blot **(A). (B)** The proliferation of MHCC97L-HOXB7 pCDNA3 cells was significantly greater than that of MHCC97L-pcDNA3 cells at 48 and 72 hours after seeding. **(C)** Compared with the control group, HOXB7 overexpression caused S phase acceleration (27.390±3.222% vs. 33.910±1.476%), and G0/G1 phase arrest (55.327±1.183% vs. 49.417±0.660%). **(D)** Wound repair assays revealed a significant increase in the wound closure rate of MHCC97L-HOXB7 pCDNA3 cells compared with control cell lines at 48 and 72 hours. **(E)** *In vitro* migration and invasion assays showed that cell migration and invasion was significantly higher in the MHCC97L-HOXB7 pCDNA3 group than in the control group at 48 and 72 hours respectively. (*, $P<0.05$; **, $P<0.01$).

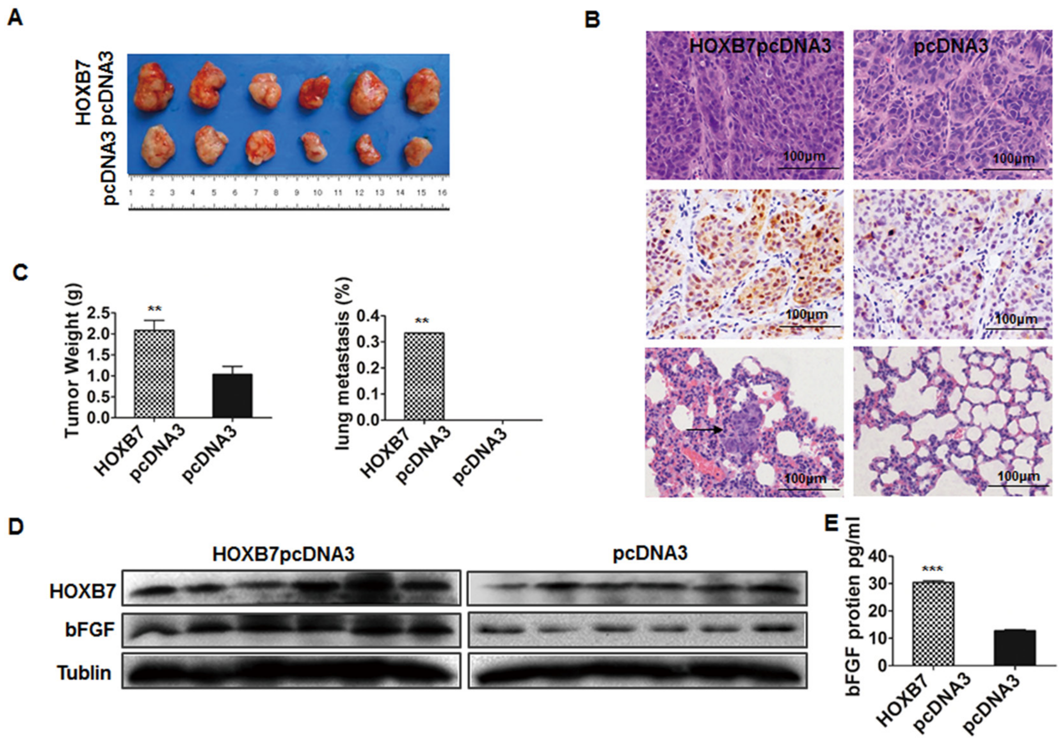

Supplementary Figure 2: Effects of up-regulation of HOXB7 on tumor growth and metastasis *in vivo*. **(A)** Morphologic characteristics of tumors in MHCC97L-HOXB7 pCDNA3 and control groups. **(B)** Representative images of tumor specimens, HOXB7 expression by immunohistochemistry and lung metastases by H&E in MHCC97L-HOXB7 pCDNA3 and MHCC97L-pcDNA3 groups. **(C)** There were significant differences in tumor weight (2.083±0.581 g vs. 1.033±0.476 g, respectively) and lung metastasis rate (30% vs. 0%, respectively) between the two groups. **(D)** MHCC97L-HOXB7 pCDNA3 group showed increased expression of HOXB7 and bFGF protein compared with control. **(E)** Serum of the MHCC97L-HOXB7 pCDNA3 group contained higher levels of bFGF protein than the control group by ELISA. (**, P<0.01; ***, P<0.001).

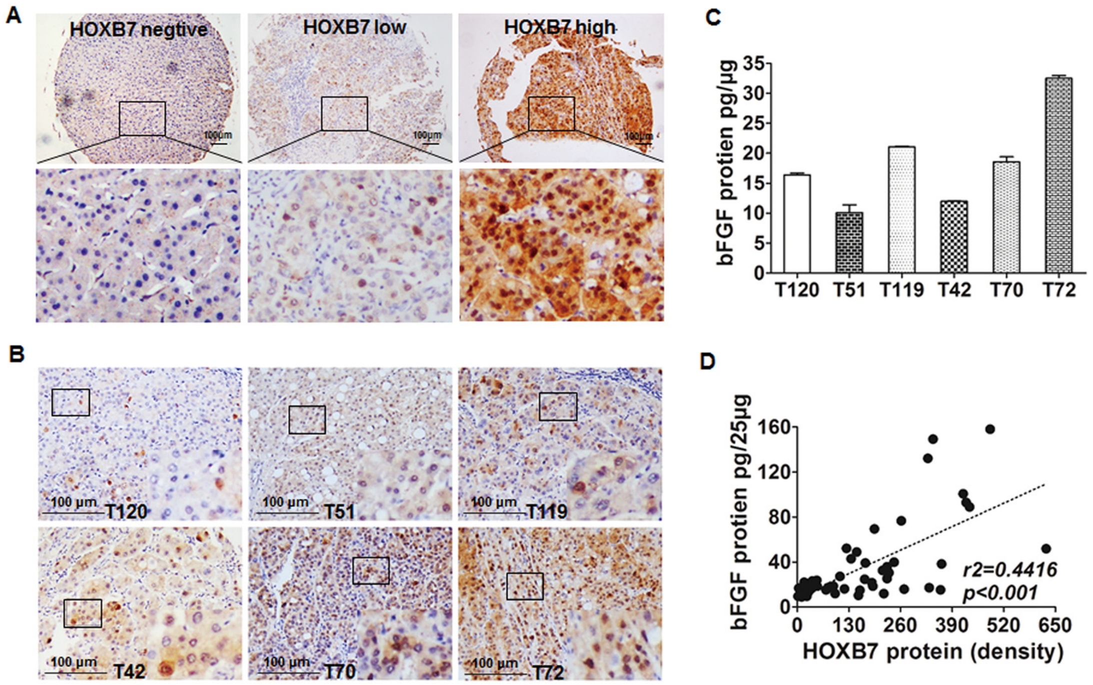

Supplementary Figure 3: Typical expression of HOXB7 in cancerous tissue by immunochemical analysis in 394 patients **(A),** in 50 cancerous tissues **(B)** and corresponding bFGF expression by ELISA **(C).** Scatter plot of HOXB7 and bFGF revealed a significant positive correlation in 50 cancerous tissues **(D)**.

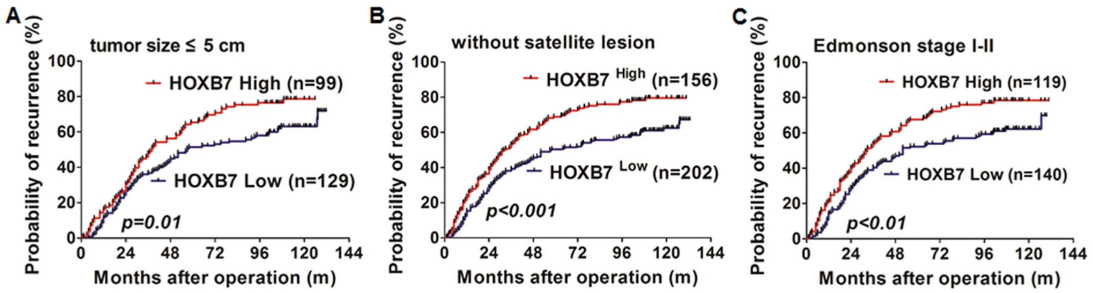

Supplementary Figure 4: Prognostic role of HOXB7 in patients with tumor size ≤5 cm **(A),** without satellite lesion **(B)** and Edmonson Stage I-II **(C).**

Supplementary Table 1: The Most Possible 10 Pathways Related to HOXB7 Based on the Databases KEGG, Biocarta, and GenMAPP, Respectively

	Pathway name	Count	P value	Q value
KEGG	MAPK signaling pathway	12	1.30E-09	1.29E-08
	p53 signaling pathway	8	8.29E-09	5.46E-08
	Focal adhesion	9	5.99E-07	1.85E-06
	Wnt signaling pathway	8	6.56E-07	1.92E-06
	Melanoma	6	1.08E-06	2.94E-06
	Bladder cancer	5	1.57E-06	3.91E-06
	Cell cycle	7	1.58E-06	3.91E-06
	Small cell lung cancer	6	3.34E-06	6.76E-06
	Pyrimidine metabolism	6	5.27E-06	9.91E-06
	Regulation of actin cytoskeleton	8	9.56E-06	1.72E-05
Biocarta	Cell Cycle: G1/S Check Point	5	1.05E-07	4.13E-07
	p53 Signaling Pathway	4	6.07E-07	1.85E-06
	Cyclins and Cell Cycle Regulation	4	3.83E-06	7.46E-06
	Influence of Ras and Rho proteins on G1 to S Transition	4	3.83E-06	7.46E-06
	Ghrelin: Regulation of Food Intake and Energy Homeostasis	3	2.78E-05	4.06E-05
	Cadmium induces DNA synthesis and proliferation in macrophages	3	4.39E-05	5.88E-05
	Hypoxia and p53 in the Cardiovascular system	3	6.51E-05	8.17E-05
	TPO Signaling Pathway	3	1.08E-04	1.26E-04
	PDGF Signaling Pathway	3	1.89E-04	1.94E-04
	EGF Signaling Pathway	3	2.14E-04	2.14E-04
GenMAPP	extracellular space	20	3.51E-18	2.77E-16
	cell growth	11	1.33E-11	4.20E-10
	regulation of cell size	11	1.33E-11	4.20E-10
	growth	11	1.13E-10	2.23E-09
	cellular morphogenesis	11	2.30E-10	3.63E-09
	regulation of growth	9	8.97E-10	1.18E-08
	Organs	12	1.29E-09	1.29E-08
	Hs_Smooth_muscle_contraction	10	3.65E-09	3.20E-08
	Hs_MAPK_signaling_pathway_KEGG	10	5.27E-09	4.16E-08
	cell-cell signaling	12	7.04E-09	5.06E-08

GenMAPP, Gene Map Annotator and Pathway Profiler; KEGG, Kyoto Encyclopedia of Genes and Genomes.

Supplementary Table 2: Primer Pairs used for qRT-PCR studies

Gene symbol	Sequence 5'-3'
HOXB7	Forward: 5'-TGGATGCGAAGCTCAGGAAC-3'<br>Reverse: 5'-GCGTCAGGTAGCGATTGTAGTG-3'
GAPDH	Forward: 5'-CA AA TTCCATGGCACCGTCAAG-3'<br>Reverse: 5'-CATCAGCAGAGGGGGCAGAGA-3'
E-cadherin	Forward: 5'-AGCCCCGCCTTATGATTCTCTG-3'<br>Reverse: 5'-TGCCCCATTCGTTCAAGTAGTCAT-3'
Ki67	Forward: 5'-AAAGAAGAGCCCCTAGCAGTCAGC-3'<br>Reverse: 5'-ACGGGCCTTTTCCTTACGAGTTC-3'
Cyclin E1	Forward: 5'-CTTCGGCCTTGTATCATTTCTCGTC-3'<br>Reverse: 5'-TTTCTTTGCTCGGGCTTTGTCC-3'
YWHAQ	Forward: 5'-TGGGGCTTGCTCTTAACTTTTCTG-3'<br>Reverse: 5'-CCTTCTGCCGCATCACATTCTT-3'
Beta-catenin	Forward: 5'-ACCAGCCGACACCAAGAAGC-3'<br>Reverse: 5'-GCGGGACAAAGGGCAAGAT-3'
ITGA10	Forward: 5'-CATGGGGGCAATTACTTCCTATCA-3'<br>Reverse: 5'-TGCTCCCATTCAGTCTGTTTGTGT-3'
IL1R2	Forward: 5'-AGGGGGACCACTCACTTACTCGT-3'<br>Reverse: 5'-GGGGGAAATGATCACAGGAATG-3'
ITGAV	Forward: 5'-CTCTCGGGACTCCTGCTACCTC-3'<br>Reverse: 5'-ACCTGCCCTCCTTCCACAATC-3'
CDK6	Forward: 5'-TGCATCGCGATCTAAAACCACA-3'<br>Reverse: 5'-CAAATATGCAGCCAACACTCCAGA-3'
PCNA	Forward: 5'-TGAAGCACCAAACCAGGAGAA-3'<br>Reverse: 5'-GCACAGGAAATTACAACAGCATC-3'
CDKN1A	Forward: 5'-GCAGGGGACAGCAGAGGAAGAC-3'<br>Reverse: 5'-CCGGCGTTTGGAGTGGTAGAA-3'
MAPK10	Forward: 5'-GGAGAATCGGCCCAAGTATGC-3'<br>Reverse: 5'-GATGCTGTAAGGCGTCGTCCAC-3'
MAP3K5	Forward: 5'-GGCACGTGCTTCCTGATGACA-3'<br>Reverse: 5'-TCTCCCCTTCTCTTCGGTAATGGT-3'
PAK3	Forward: 5'-CATTGGCCCCTCCTGTGTCTG-3'<br>Reverse: 5'-TTCTTTGCCGATCTGTGTTCCTGT-3'
FGF2	Forward: 5'-CACTTCAAGGACCCCAAGAG-3'<br>Reverse: 5'-GAAGCACTCGTCAGTAACACAT-3'

qRT-PCR, quantitative real time reverse transcription polymerase chain reaction; F, forward; R, reverse.

Supplementary Table 3: Primer Pairs used for CHIP

Basic FGF promoter region	Sequence 5’-3’
1	Forward: 5’-GAGATACTATTATACAGTTTTG-3’<br>Reverse: 5’-TGGTCTCAAACTCCTGGGGTCA-3’
2	Forward: 5’-TGACCCCAGGAGTTTGAGACCA-3’<br>Reverse: 5’-AATAGATGTCAAAATGAAAATG-3’
3	Forward: 5’-GAAGATACAATTCATGTATCAT-3’<br>Reverse: 5’-ATGATACATGAATTGTATCTTC-3’
4	Forward: 5’-GAAGATACAATTCATGTATCAT-3’<br>Reverse: 5’-TAACCAAAATGTGGAAACAAGC-3’
5	Forward: 5’-GCTTGTTTCCACATTTTGGTTA-3’<br>Reverse: 5’-TAATCTAATTTTCAAATGGGAA-3’
6	Forward: 5’-TTCCCATTTGAAAATTAGATTA-3’<br>Reverse: 5’-TGAAGGTCTTATGGTCTGCCTC-3’
7	Forward: 5’-GAGGCAGACCATAAGACCTTCA-3’<br>Reverse: 5’-TTGGTATATGTTCTATAGAGGA-3’
8	Forward: 5’-TCCTCTATAGAACATATACCAA-3’<br>Reverse: 5’-TTCTTAAGTCTCCAAGAAGGCT-3’
9	Forward: 5’-AGCCTTCTTGGAGACTTAAGAA-3’<br>Reverse: 5’-ATTGTTATGCATTTCTTGGAGA-3’
10	Forward: 5’-GATTTAGAGATTTTCAAAGCCT-3’<br>Reverse: 5’-AGTTCTCCTCCCTCCTGCGCGC-3’

Supplementary Table 4: The Clinicopathologic Characteristics of HCC Patients

Clinical and pathological indexes		Original cohort 1 (n=394)		Original cohort 2 (n=50)	
		Number	Percentage (%)	Number	Percentage (%)
Age (years)	>50	194	49.2	27	54.0
	≤50	200	50.8	23	46.0
Sex	Male	344	87.3	47	94.0
	Female	50	12.7	3	6.0
AFP (ng/ml)	>20	253	64.2	30	60.0
	≤20	141	35.8	20	40.0
HBsAg	Positive	323	82.0	45	90.0
	Negative	71	18.0	5	10.0
HCV	Positive	8	2.0	0	0.0
	Negative	386	98.0	50	100.0
GGT (U/l)	>54	231	58.6	28	56.0
	≤54	163	41.4	22	44.0
ALT (U/l)	>75	37	9.4	8	16.0
	≤75	357	90.6	42	84.0
Liver cirrhosis	Yes	320	81.2	37	74.0
	No	74	18.8	13	26.0
Tumor size (cm)	>5	166	42.1	17	34.0
	≤5	228	57.9	33	66.0
Tumor number	Multiple	79	20.1	4	8.0
	Single	315	79.9	46	92.0
Vascular invasion	Yes	98	24.9	21	42.0
	No	296	74.1	29	58.0
Tumor	None	163	41.4	33	66.0
encapsulation	Complete	231	58.6	17	34.0
Edmondson Stage	III-IV	135	34.3	21	42.0
	I-II	259	65.7	29	58.0
Satellite lesion	Yes	36	9.1	7	14.0
	No	358	90.9	43	86.0
Child-Pugh Stage	B	19	4.8	0	0.0
	A	375	95.2	100	100.0
BCLC Stage	B+C	87	22.1	10	20.0
	0+A	307	77.9	40	80.0

Abbreviations: AFP, α-fetoprotein; HBsAg, hepatitis B surface antigen; HCV, hepatitis C virus; GGT, γ-glutamyl transpeptidase; ALT, alanine aminotransferase; BCLC, Barcelona Clinic Liver Cancer.
